# Bulle intra-cristallinienne après chirurgie du décollement de rétine rhématogène par voie externe

**DOI:** 10.11604/pamj.2015.22.327.8464

**Published:** 2015-12-03

**Authors:** Rajaa Elhannati, Hicham Tahri

**Affiliations:** 1 Service D'ophtalmologie, CHU Hassan II, Fès, Maroc

**Keywords:** Cristallin, tamponnement interne, décollement de rétine, lens, internal tamponade, retinal detachment

## Image en medicine

Il s'agit d'une patiente âgée de 48 ans connue forte myope opérée pour décollement de rétine rhégmatogène de l'œil gauche sur une déchirure supérieure à 1H avec soulèvement maculaire et PVR stade B dans un délai de 15 jours. La chirurgie par voie externe a consisté en une rétinopexie par cryoapplication, une indentation radiaire et un tamponnement interne par une injection intravitréenne du gaz SF6. Les suites postopératoire sont marquées par une amélioration de l'acuité visuelle à 1/10ème avec une ré-application de la rétine au pôle postérieur. Par ailleurs on a noté la présence d'une bulle intra-cristallinienne entrainant une opacification du cristallin localisée (A, B, C).

**Figure 1:**
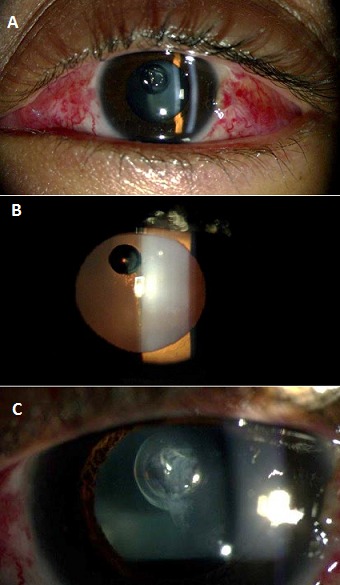
(A) image de la lampe à fente de face montrant la bulle intra-cristallinienne après chirurgie du décollement de rétine par voie externe; (B) image en rétro-illumination montrant la bulle intra-cristallinienne avec un cristallin clair sur le reste; (C) image en grossissement à la lampe à fente montrant la présence d'une opacification localisée au niveau de la bulle intra-cristallinienne

